# Impact of Triacetin- and Tributyrin-Based Formulations on IBS Microbiome Composition and SCFA Profiles: A Preclinical Study

**DOI:** 10.3390/ijms27198910

**Published:** 2026-10-07

**Authors:** Liam Jenkins Sánchez, Jonas Poppe, Aurélien Baudot, Lam Dai Vu, Will Villella, Pieter Van den Abbeele

**Affiliations:** 1 Cryptobiotix SA, Technologiepark-Zwijnaarde 82, 9052 Ghent, Belgiumjonas.poppe@cryptobiotix.com (J.P.); aurelien.baudot@cryptobiotix.com (A.B.); lamdai.vu@cryptobiotix.com (L.D.V.); 2 Gundry MD^®^, 145 S. Fairfax Avenue, Suite 200, Los Angeles, CA 90036, USA; will.villella@gundrymd.com

**Keywords:** triacetin, tributyrin, irritable bowel syndrome (IBS), Systemic Intestinal Fermentation Research (SIFR^®^), microbiome, SCFAs, metagenomics

## Abstract

Irritable bowel syndrome (IBS) is a prevalent yet poorly understood functional gut disorder frequently associated with microbial dysbiosis, making microbiome-targeting interventions a promising management strategy. In this study, we evaluated the microbiome-modulating potential of three doses of triacetin (TA; ReBiome™; 100, 200 and 300 mg/day) and two finished formulations containing TA and/or tributyrin (TB): Active Biome™ (AB) and Bio Complete 3™ (BC3). Inulin served as a benchmark prebiotic. The validated ex vivo Systemic Intestinal Fermentation Research (SIFR^®^) technology pipeline was applied using fecal microbiota from eight adults with IBS. TA dose-dependently increased short-chain fatty acid (SCFA) levels from 100 mg/day, significantly increasing acetate and butyrate while maintaining low gas formation. In addition to acetate and butyrate, AB and BC3 also promoted propionate while BC3 induced a particularly pronounced butyrate increase. Overall, both formulations induced stronger SCFA but milder gas increases than inulin. TA, AB and BC3 enriched IBS-depleted, butyrate-associated OTUs related to *Faecalibacterium* and *Anaerobutyricum* species. AB and BC3 increased microbial diversity (community modulation score (CMS)) by promoting Bifidobacteriaceae, Lachnospiraceae and Bacteroidaceae. Overall, TA and TA/TB-containing formulations promoted butyrate production, potentially helping mitigate IBS symptom severity by inducing a favorable fermentation profile with limited gas formation.

## 1. Introduction

Irritable bowel syndrome (IBS) is a disorder of gut function, characterized by recurrent abdominal pain and altered stool frequency or consistency [1]. While exhibiting a high prevalence of 5–10% of otherwise healthy individuals, its etiology remains poorly understood and likely involves multiple interacting disturbances, such as alterations of gut motility, visceral sensitivity, epithelial barrier function, immune pathways and/or gut–brain signaling [1,2]. Emerging evidence implicates the gut microbiota, although the substantial heterogeneity among patients and studies has precluded the identification of a single universal IBS-associated microbial signature [2].

Altered microbial metabolism may nevertheless contribute to the clinical features of IBS. The gut microbiota is known to produce important metabolites which participate in host–microbial communication, chief among them short-chain fatty acids (SCFAs) including acetate, propionate and butyrate [3,4]. Importantly, butyrate is the main energy source for colonocytes [5] and is thus key to maintaining gut barrier integrity [6], a salient deficiency in IBS [7]. This may, at least in part, reflect the depletion of butyrate-producing taxa that has been consistently reported in IBS-associated microbiota [8]. Accordingly, interventions that restore microbial metabolic functioning may represent a potential strategy for IBS management [2]. Such interventions should be carefully designed, as microbial butyrate formation is also connected to intestinal gas production [9,10], an important exacerbating factor in IBS symptoms [11]. This generates a need for microbiome interventions that improve metabolic function without neglecting tolerability. Unfortunately, clinical evidence for microbiome-directed approaches remains inconsistent, emphasizing the need for controlled mechanistic evaluation.

We previously demonstrated that triacetin (TA, ReBiome™) beneficially modulates the IBS-derived microbiota ex vivo by increasing butyrate production with low gas formation, while promoting microbial changes associated with improved gut barrier integrity in a cell-based model [12]. Promisingly, tributyrin (TB), a glycerol triester of butyrate, has been shown to increase intestinal butyrate availability and alleviate gut dysbiosis in mouse models [13,14]. These findings suggest that TA and TB-based formulations may simultaneously deliver SCFAs without excessive gas formation, while favorably modulating the microbiome composition. Therefore, they represent promising microbiome-directed strategies for restoring microbial function in IBS [12].

Building on these findings, we investigated two commercially available formulations incorporating TA and/or TB: Active Biome™ and Bio Complete 3™ (AB and BC3; Gundry MD^®^ Los Angeles CA, USA). Whereas TA alone has demonstrated a favorable SCFA-to-gas profile, it remains unclear whether this activity is retained when TA or TB is incorporated into complex formulations containing prebiotic and probiotic components. Evaluating the complete formulations therefore enabled us to address whether formulation context preserved the low-gas fermentation profile while broadening microbial compositional and metabolic responses.

The aim of this study was thus to investigate whether AB and BC3 could modulate the compositional and metabolic features of IBS-associated dysbiosis relative to TA alone and the benchmark prebiotic inulin. To address this research question, we employed the validated ex vivo SIFR^®^ technology platform (Ghent, Belgium). This approach preserves donor-specific microbiota under standardized fermentation conditions while enabling direct quantification of microbial composition and metabolites, including SCFAs that are rapidly absorbed (and therefore difficult to quantify) in vivo. Thus, it also provides mechanistic insights into microbiome modulation with demonstrated translational relevance to human intervention studies [15].

## 2. Results

### 2.1. IBS Donor Microbiotas Show Diverse Yet Characteristic Compositional Patterns

Exploratory compositional analysis of the microbiota of IBS donors revealed pronounced interindividual heterogeneity. While exhibiting distinct compositional features compared to healthy microbiota, IBS samples could still be classified according to the concept of enterotypes [16]. Donors 3/5 exhibited important contributions of *Prevotella* OTUs, markers of the *Prevotella* enterotype, while donors 1/2/4/8 had increased concentrations of *Bacteroides*/*Phocaeicola*, aligning with *Bacteroides*. Donors 6/7 were enriched in *Ruminococcus_E*, a marker of the *Ruminococcus* enterotype (Figure 1A).

When comparing the IBS and healthy subgroups, distinct patterns emerged. Notably, IBS-associated microbiota was enriched in OTUs belonging to *Blautia_A* while being depleted in *Fusicatenibacter* taxa. Tendencies were observed for a depletion in *Faecalibacterium* and *Bifidobacterium* OTUs, but these were not statistically significant due to high inter-donor compositional variation (e.g., IBS donor 3 exhibited high *Bifidobacterium* loads, Figure 1B).

Altogether, these findings indicate that the fecal microbiota of the eight IBS adult donors captured broad compositional diversity and phenotype-specific variation observed in vivo, strengthening the robustness of our findings.

### 2.2. TA Enhanced SCFA Production from the Lowest Dose, While AB and BC3 Promoted Distinct SCFA Profiles with Limited Gas Formation

As expected, the established prebiotic IN was well-fermented, leading to the production of SCFAs (acetate, propionate and butyrate), with concomitant acidification and gas formation (Figure 2).

TA likewise modulated fermentation parameters in a dose-dependent manner. Notably, statistically significant impacts were observed starting at the lowest dose TA_L, equivalent to only 100 mg/day. TA selectively increased acetate and butyrate, resulting in higher total SCFA concentrations at all tested doses. At equivalent doses of 300 mg/day, these effects exceeded those of IN, with TA_H inducing significantly greater stimulation of fermentative parameters. This SCFA-promoting activity remained dose-effective across all three tested concentrations, with especially favorable responsiveness for butyrate (Figure S1). Further, TA did not significantly impact propionate at any dose (Figure 2C–F). These metabolic changes were accompanied by dose-dependent acidification, while importantly gas production remained limited and significantly below that of IN even at the highest dose TA_H (Figure 2A,B).

AB and BC3 elicited fermentation responses broadly comparable to TA_H, particularly with respect to pH, gas production and total SCFA levels. Whereas AB largely resembled TA_H by predominantly stimulating acetate, BC3 selectively increased butyrate levels beyond the effect of all other products, with only slight effects on acetate. Thus, the characteristic SCFA-enhancing profile of TA was retained following its incorporation into AB, while BC3 achieved a comparable overall fermentation profile through a distinct, butyrate-dominant response.

While higher SCFA levels are often coupled with increased gas formation, a lower gas/SCFA ratio is desirable in regard to in vivo tolerability while still exerting potential health benefits. In line with expectations, IN increased SCFA production with a proportional increase in gas levels. In contrast, TA dose-dependently boosted SCFAs while gas levels remained moderate, resulting in a lower and progressively decreasing gas/SCFA ratio as TA doses increased (Figure 3). This observation was significant at the lowest tested dose (TA_L). Additionally, AB and BC3 exhibited gas-to-SCFA ratios comparable to TA_H, indicating that both complete formulations combined robust SCFA production with relatively limited gas formation (Figure 3).

### 2.3. TA, AB and BC3 Differentially Modulated IBS Donor-Derived Microbiota and Promoted SCFA-Associated Taxa

The divergent fermentation profiles were mirrored by distinct alterations in microbiota composition. First, divergent shifts were observed on overall taxonomic diversity. The community modulation score (CMS) was decreased by IN (not significant) whether measured at the OTU or genus level, reflecting uneven growth of specific taxa. In contrast, all TA doses, BC3 and especially AB significantly increased the CMS, implying broad stimulation of numerous genera and OTUs (not significant for TA_L at the genus level, Figure 4A,B). Traditional indices of taxonomic richness (Chao1) showed no significant effects for any of the products, while those for evenness (Shannon and reciprocal Simpson indices) were significantly decreased by IN and BC3, implying specific stimulation of certain taxa by these compounds (Figure S2).

Compositional analysis at the family level revealed diverse stimulatory patterns by the products, with IN stimulating Bifidobacteriaceae, Coprobacillaceae and to a lesser extent Lachnospiraceae, while suppressing Aristaeellaceae along with various other families (not significant). TA broadly promoted several SCFA-associated families, with effects scaling according to the employed dose. Most prominently, stimulation was observed on Lachnospiraceae (at all doses), Ruminococcaceae (only significant for TA_M), Rikenellaceae (only significant for TA_M) and at higher doses Eggerthellaceae and Acutalibacteraceae (only for TA_H). In contrast, Bifidobacteriaceae was slightly repressed by TA_H, while decreases in Aristaeellaceae and Akkermansiaceae were noted yet non-significant. AB and BC3 built on the effects of TA_H, with important additional modulations. AB more strongly stimulated Ruminococcaceae and boosted Bacteroidaceae. BC3, additionally to the effects of AB, increased levels of Coprobacillaceae but also significantly suppressed Aristaeellaceae, Rikenellaceae and Akkermansiaceae (Figure 4C).

Specific bacterial families correlated with levels of certain SCFAs, linking compositional and metabolic shifts. Notably, Lachnospiraceae abundance correlated with butyrate levels (Figure 4D) while Bacteroidaceae correlated with propionate formation (Figure 4E).

Investigation at OTU-level resolution revealed further product effects, including the well-established bifidogenic effect of IN (Figure 5A). Administration of TA tended to promote butyrate-related OTUs within Ruminococcaceae (*Faecalibacterium butyricigenerans*/*prausnitzii*) and Lachnospiraceae (*Anaerobutyricum hallii*/*soehngenii*, *Bariatricus comes* and *Mediterraneibacter_A butyricigenes*, Figure 5B). AB exerted wide-reaching effects beyond those of TA, also promoting *Bifidobacterium* OTUs (*B. adolescentis*/*faecale* and *B. longum*) as well as further taxa within Lachnospiraceae (*Agathobacter rectalis*, *Coprococcus_A catus*) and Bacteroidaceae (*Phocaeicola dorei*, *Bacteroides thetaiotaomicron*, *Bacteroides uniformis*, Figure 5C). In contrast, BC3 showed a distinct profile from AB, selectively elevating *Bifidobacterium bifidum* only among Bifidobacteriaceae, and strongly stimulating *Bacteroides uniformis* in place of *B. thetaiotaomicron*. While also promoting the same butyrate-related taxa as AB, BC3 stimulation of *Bariatricus comes* and *Mediterraneibacter_A butyricigenes* was not significant. A full heatmap representation of the OTUs significantly impacted by the products can be found in the Supplementary Materials (Figure S3).

## 3. Discussion

The aim of this work was to investigate the potential of triacetin (TA, ReBiome™) and TA and/or tributyrin (TB)-containing products Active Biome™ and Bio Complete 3™ (AB and BC3) to favorably reshape the microbiome of IBS subjects. For this purpose, a validated ex vivo approach using SIFR^®^ technology was employed, using inulin (IN) as a prebiotic comparator. Consistent with previous reports, the IBS-related microbiota of eight heterogeneous IBS subjects presented elevated *Blautia_A* OTUs, while the genera *Bifidobacterium*, *Faecalibacterium* and *Fusicatenibacter* tended to be underrepresented. Promisingly, TA, AB and BC3 elicited favorable metabolic profiles, while supporting the growth of IBS-depleted gut microbes.

TA, AB and BC3 all increased overall SCFA levels, albeit with different composition patterns. Promisingly, gas formation remained limited. This contrasts with proportionally higher gas release observed upon fermenting the well-established prebiotic IN. Such an activity profile is of special interest in an IBS context, as intestinal gases are known to exacerbate symptoms [17]. By selectively promoting SCFA production while limiting gas generation, TA, AB and BC3 exhibited a fermentation profile of potential relevance to IBS, potentially supporting further investigation of these products in vivo. Indeed, low-FODMAP diets have proven clinically effective for symptom control, indicating that microbiome-targeting approaches should ideally provide benefits in a compatible manner [18]. Based on our results, TA, AB and BC3 may prove to be promising interventions for increasing SCFA levels with limited gas formation in colonic contexts [12].

Notably, the magnitude of the SCFA-boosting effect from TA went beyond delivery of acetate, as total SCFA amounts were larger than theoretical acetate equivalents would suggest. Indeed, dosing TA at 100, 200 and 300 mg/day resulted in 104.3%, 104.0% and 114.3% of the dosed carbon as SCFAs respectively, consistent with our previous work which returned 123.2% from 400 mg/day of TA [12]. Such results imply active alternative SCFA-producing pathways, such as fermentation of the glycerol backbone, stronger acidification and/or the modulation of activity by the gut microbiota [12]. Microbial compositional analysis appeared to support this last mechanism. TA and TB-containing products successfully modulated gut microbial ecology, as captured by the community modulation score (CMS). By this measure, which avoids the bias introduced by cell density increases caused by prebiotics [19], TA, AB and BC3 increased microbial diversity by boosting a large array of gut microbes, unlike IN which showed narrow effects. Simultaneously, shifts in microbial composition by TA, AB and BC3 crucially tended to boost butyrate-producing taxa. Among these, *Faecalibacterium butyricigenerans*/*prausnitzii* stimulation was especially relevant, as a major butyrate producer of the gut microbiota [20] capable of cross-feeding with acetate, usually mediated via bifidobacteria [21]. Additionally, its depletion in IBS is well-recorded [2,22], making its stimulation potentially relevant in the context of IBS-associated dysbiosis. Likewise, the abundance of *Anaerobutyricum hallii*/*soehngenii*, a butyrate-producing OTU linked to improved metabolic health [23], was elevated by TA, AB and BC3. These compositional shifts are consistent with enhanced microbial conversion of acetate into butyrate, providing a plausible explanation for the SCFA profiles observed following the treatments. Given that butyrate is the primary energy source for colonocytes [5] and plays a key role in maintaining epithelial barrier integrity [6], the observed increase in butyrate production may be physiologically relevant. Indeed, as an impaired gut barrier is a hallmark of IBS, increasing butyrate production may target a central disease-associated mechanism [7].

By testing different doses of TA, we were able to gain insights into its dose-responsiveness, which is relevant for potential product development. Notably, we observed significant effects by TA starting at the lowest dose of only 100 mg/day. This suggests that measurable effects on microbial metabolism may occur with low-dose supplementation. Furthermore, increases in concentration resulted in scaling effects up to 300 mg/day. Uniquely to the standalone TA doses, the butyrate-producing OTUs *Bariatricus comes* and *Mediterraneibacter_A butyricigenes* were promoted, a finding consistent with previous work [12]. These taxa potentially also contribute to the conversion of administered acetate into butyrate.

Importantly, AB exhibited an SCFA-enhancing, low-gas fermentation profile broadly comparable to that observed with TA_H. As AB contained the same amount of TA as TA_H (300 mg/day), the standalone TA treatment provides useful context for interpreting the formulation-level response, although the contribution of TA cannot be separated from those of the other AB components or their interactions. It is nevertheless consistent with our previous observations of the effects of TA [12]. BC3, which contained TB rather than TA, exhibited a similarly favorable gas-to-SCFA profile but produced a distinct, butyrate-dominant response. Overall, AB and BC3 induced broader microbiome responses than TA, aligning with their composition combining TA and/or TB with additional functional ingredients, including prebiotic matrices and probiotics. Both formulations stimulated additional Lachnospiraceae taxa, including *Coprococcus*_A *catus* and *Agathobacter rectalis*, both butyrate-producing taxa with anti-inflammatory properties [24,25]. In contrast to the TA doses, both AB and BC3 increased levels of *Bifidobacterium* OTUs. For BC3-treated samples, this reflected successful colonization by the probiotic OTU *B. bifidum*. Support for *Bifidobacterium* is of clinical relevance as this genus is commonly depleted in IBS [26] and plays a key role in cross-feeding interactions supporting beneficial secondary fermenters [27,28]. Unlike TA, AB and BC3 also significantly increased propionate levels. This coincided with divergent stimulation of propionogenic taxa, namely *Phocaeicola dorei* [4] and *Bacteroides* [29] OTUs by AB, and *Bacteroides uniformis* [30] selectively by BC3. Propionate carries health benefits in vivo, such as inducing satiety and improving weight management [31,32], suggesting the more complete SCFA profile induced by AB and BC3 may carry additional benefits compared to TA.

The activity of TB warrants further discussion, especially in the context of BC3. First, unlike TA, TB was not evaluated as a standalone control. Consequently, the contributions of TB to the responses elicited by BC3 cannot be distinguished. Nevertheless, BC3, the finished product containing large amounts of TB, produced the largest increase in butyrate among the products. While direct hydrolysis of TB may contribute to this response [33], the absence of a TB control precludes attribution of this effect specifically to TB. Moreover, the accompanying compositional changes suggest that the response to BC3 was not limited to direct butyrate delivery. BC3 also increased acetate concentrations despite the lack of TA in this formulation, likely via elevated *B. bifidum* [34], potentially providing precursors for butyrate formation. Together with the stimulation of multiple butyrate-producing taxa, this suggests BC3 may induce a targeted remodeling of the microbiome towards enhanced butyrate formation.

Several limitations must be acknowledged. This study was based on fecal microbiota samples derived from eight IBS subjects. While a broadly representative diversity of compositional profiles was observed, any extrapolation to the population-level implications must be approached cautiously and interpreted as exploratory. Dietary information on the donors was also not available, which may influence interindividual differences in baseline microbiota, and thus product response. Furthermore, IBS-C subjects were not included in the study, in order to prevent differences in microbial composition due to longer colonic transit times from confounding results. As such, the effects of the products in this IBS subtype remain to be established. The possible beneficial activity of the products on gut-barrier function was also not directly tested in this study, although beneficial activity of TA [12] and butyrate [6] has been established previously. Finally, these results rely on preclinical colonic simulations performed ex vivo. Although validated experimentally [15], conclusions on possible health effects will require additional investigations as the present study did not assess host responses, clinical symptoms or tolerability. Follow-up studies with larger sample sizes are needed to confirm the health-promoting and clinical efficacy of these formulations, such as host–microbiome interaction assays to validate the effects on gut integrity [12,34], or, ideally, large-scale clinical trials.

Overall, these findings shift the perspective on TA and TA/TB-containing formulations from simple SCFA-delivery compounds towards modulators of the gut microbial ecosystem. The observation that AB exhibited a low-gas fermentation profile comparable to standalone TA suggests that TA activity is sufficiently robust to be retained within a complex finished formulation, while the distinct response elicited by BC3 illustrates how glycerol ester identity and broader formulation context can further shape microbial metabolism. More broadly, the results support the concept that carefully designed glycerol ester-based formulations may help restore metabolic functions that are impaired in IBS-derived microbiota without proportionally increasing gas formation that can aggravate symptoms. Rather than merely supplying acetate or butyrate, such formulations may therefore offer a means of steering the microbiome towards more favorable metabolic outputs.

## 4. Materials and Methods

### 4.1. Test Products

Two complex formulation products, Active Biome™ and Bio Complete 3™ (AB and BC3; Gundry MD^®^ Los Angeles CA, USA), were tested alongside triacetin (TA; ReBiome™, Compound Solutions, Carlsbad, CA, USA), inulin (IN; derived from chicory, I2255, Merck, Overijse, Belgium) and a no-substrate control (NSC) without any added products.

Human-equivalent doses for AB and BC3 were 700 and 600 mg/day respectively, corresponding to a recommended intake of 2 capsules per day. At these doses, AB consisted of 300 mg triacetin and 50 mg tributyrin combined with a prebiotic blend (acacia gum and a polyphenol fiber matrix of apple, carrot, blueberry, cranberry and spinach fiber), a polyphenol mix (*Dioscorea alata* root, *Vitis vinifera* seed extract) and 10 mg of microencapsulated *Akkermansia muciniphila*, while BC3 contained 300 mg tributyrin, a prebiotic matrix (acacia and partially hydrolyzed guar gum) and 22 mg of a blend of *Bacillus coagulans* and *Bifidobacterium bifidum*. TA was tested at three different concentrations of 100, 200 and 300 mg/day (TA_L, TA_M and TA_H respectively). Inulin was added as a reference prebiotic comparator at 300 mg/day.

### 4.2. Donor Selection

To investigate the effects of AB, BC3, TA and IN on the microbiota of IBS subjects ex vivo, cryopreserved fecal material was used, which was derived from eight donors (age = 43.8 ± 6.3 years). Subjects presented either IBS-M (mixed type) or IBS-D (diarrhea type) as defined by Rome IV criteria [35], while IBS-C patients (constipation type) were excluded to prevent increased colonic transit time from acting as a confounding factor in microbiota composition [36]. Individuals with a BMI over 25 or under 20 were likewise excluded due to expected differences in microbial profiles [37]. Furthermore, the following criteria were applied: age 30–65, no antibiotic/probiotic intake in the 3 months prior to study, no other gastrointestinal disorders (cancer, ulcers, IBD), non-smoking and alcohol consumption under 3 units per day. No food diaries were collected.

To establish whether the test subjects displayed a gut microbiota profile characteristic of IBS-linked dysbiosis, comparative fecal samples were sourced from eight healthy adults (age = 36.7 ± 5.9 years) not presenting IBS. These healthy adults otherwise complied with the same criteria as the IBS subjects.

### 4.3. Ex Vivo Colonic Fermentations Using the SIFR^®^ Technology Platform

Colonic fermentations were performed as previously described [15]. Fecal slurries were prepared from cryopreserved fecal material by mixing in an anaerobic phosphate buffer (0.05 M). Subsequently, bioreactors were prepared containing 5 mL of a blend of nutritional medium (M0017, Cryptobiotix, Ghent, Belgium), fecal microbiota and relevant test products, and kept anaerobic using a bioreactor management device (Cryptobiotix, Ghent, Belgium). Reactors were incubated under constant agitation (140 rpm) at 37 °C (MaxQ 6000, Thermo Scientific, Merelbeke, Belgium).

This study consisted of 7 study arms (AB, BC3, TA_L, TA_M, TA_H, IN and NSC) that were each assessed for eight individual IBS subjects as biological replicates (*n* = 8). Analysis of key fermentative parameters and microbial composition was performed at 0 h (only for NSC) and at 24 h of fermentation (all study arms). The microbiota of the eight healthy adults was investigated for microbial composition at 0 h only (NSC).

Finally, the NSC arm was run in technical triplicates (*n* = 3) for each of the eight IBS subjects with analysis of key fermentative parameters at 24 h for the purposes of quality control (Figure 6). The coefficient of variation was 2.90%, confirming the high technical reproducibility of the SIFR^®^ technology. These two additional NSC technical replicates were not considered for statistical comparisons between the study arms.

### 4.4. Analytics of Key Fermentative Parameters

Short-chain fatty acids (SCFAs; acetate, propionate and butyrate) were determined via gas chromatography with a flame ionization detector, as previously described [38]. Briefly, 0.5 mL samples were diluted in distilled water (1:3), acidified with 0.5 mL of 48% sulfuric acid, and supplemented with an excess of sodium chloride, 0.2 mL of internal standard (2-methylhexanoic acid), and 2 mL of diethyl ether. After homogenization and phase separation, the diethyl ether layer was collected for analysis using a Trace 1300 chromatograph (Thermo Fisher Scientific, Merelbeke, Belgium) equipped with a Stabilwax-DA capillary GC column, a flame ionization detector, and a split injector using nitrogen gas as the carrier and makeup gas. The injection volume was 1 µL and the temperature ranged from 110 °C to 240 °C. The temperatures of the injector and detector were 240 and 250 °C, respectively. Sample pH was measured using an electrode (Hannah Instruments Edge HI2002, Temse, Belgium).

### 4.5. Microbial Composition

Microbial composition was analyzed via quantitative 16S rRNA gene profiling targeting the V3-V4 region. First, DNA was extracted via the SPINeasy DNA Kit for Soil (MP Biomedicals, Eschwege, Germany). Subsequently, library preparation and sequencing were performed on an Illumina MiSeq platform with v3 chemistry (2 × 300 bp). Amplicons were generated via the primers 341F (5′-CCT ACG GGN GGC WGC AG-3′) and 785Rmod (5′-GAC TAC HVG GGT ATC TAA KCC-3′). Pre-processing and OTU picking were performed with Mothur v1.35.1 [39], while the annotation of representative sequences employed NCBI blast v2.10.0 [40].

Absolute cell densities per individual operational taxonomic unit (OTU) were estimated by multiplying relative abundances (%) obtained via 16S rRNA gene sequencing by the total cell counts of each sample. Cell densities were quantified by diluting samples in PBS, staining cells with SYTO 16 and counting via a NovoCyte flow cytometer (Agilent, Santa Clara, CA, USA).

### 4.6. Data Analysis

Data analysis was performed using R (version 4.4.3, www.r-project.org, accessed on 29 June 2026). The ggplot2 package (v4.0.0) was used to make a series of violin plots, bar charts, and heatmaps. Statistical analysis was conducted using a linear mixed-effects model, which accounted for both fixed effects (treatment) and random effects (interpersonal variation), using glmmTMB package v1.1.11 [41]. The Benjamini–Hochberg correction was applied to control the false discovery rate across parameters [42], using stats package v3.6.2. Effects of products compared to the reference (NSC) were assessed via a post hoc pairwise comparison using estimated marginal means (emmeans package v1.11.2), again with the Benjamini–Hochberg correction of *p*-values. Effects were significant at *p*_adjusted_ < 0.05. In the violin plots, statistical differences between the products and the NSC are indicated with * (0.01 < *p*_adjusted_ < 0.05), ** (0.001 < *p*_adjusted_ < 0.01) or *** (*p*_adjusted_ < 0.001), while differences between the products and IN were indicated with $/$$/$$$. The community modulation score (CMS) [19] was calculated for both the OTU and genus level, and measures the balance between the number of OTUs/genera that are stimulated and those that are suppressed. Finally, pairwise correlation analysis using Spearman’s rank correlation coefficient was performed between SCFAs and absolute levels of bacterial families.

## Figures and Tables

**Figure 1:**
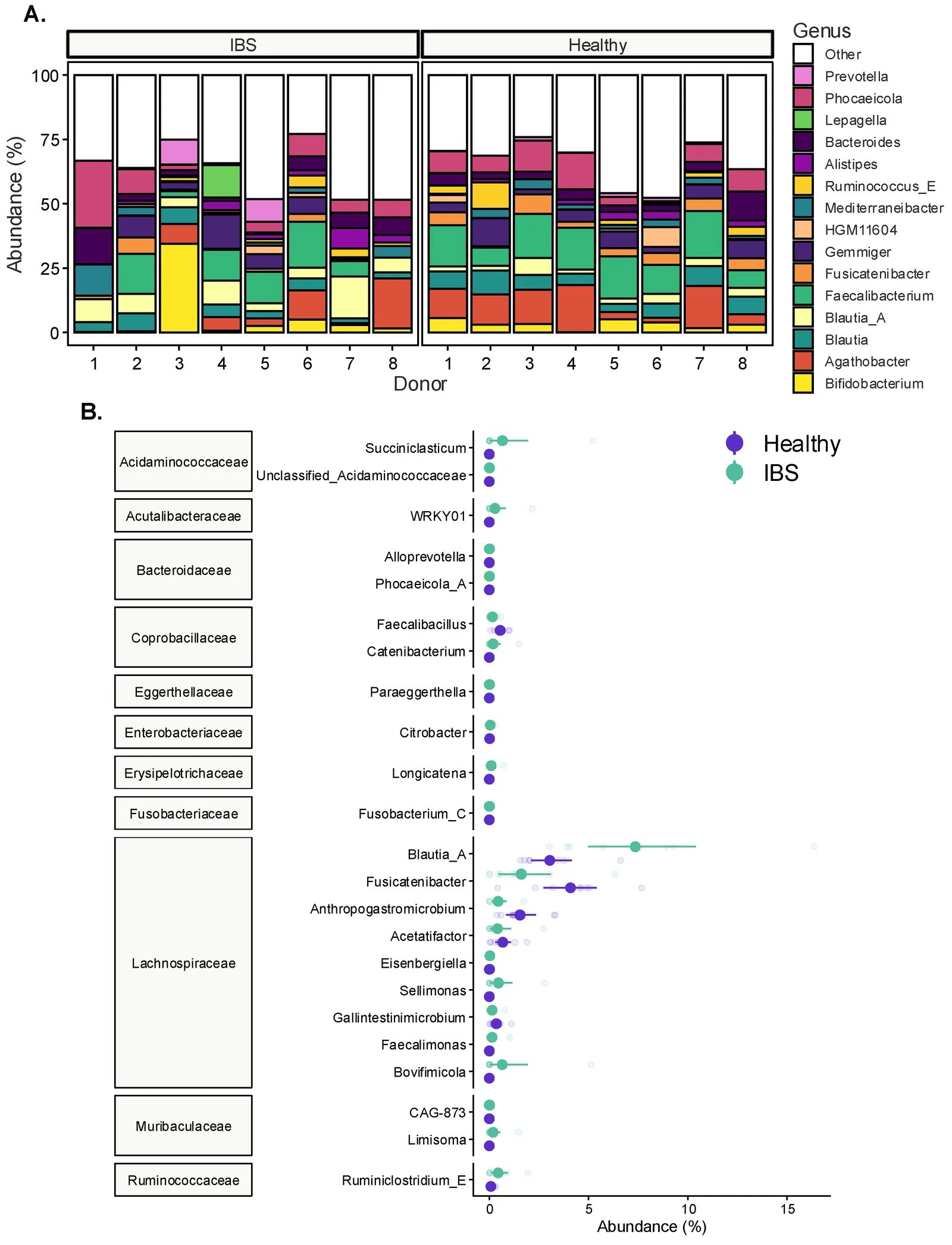
The microbiota of the various IBS subjects displayed marked heterogeneity, while showcasing IBS-associated patterns compared to a healthy cohort, at 0 h. (**A**) Abundances (%) of the most abundant genera for each of the eight IBS or eight healthy adult donors. (**B**) Bacterial taxa that displayed differences in relative abundance (%) between IBS and healthy cohorts (*p* < 0.05).

**Figure 2:**
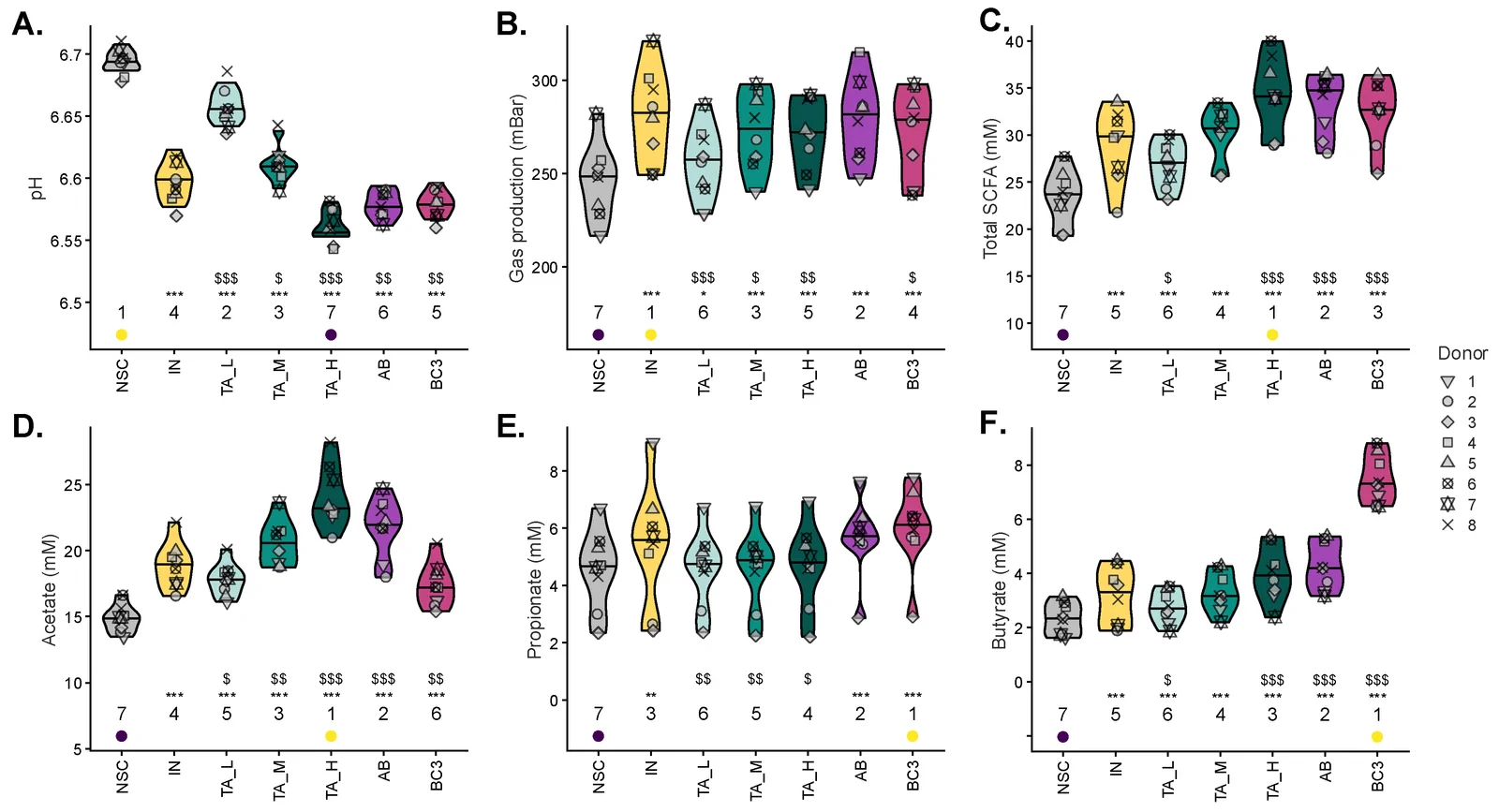
TA boosted acetate and butyrate in a dose-dependent manner, with AB additionally promoting propionate and BC3 selectively increasing butyrate levels. The impact of the products at 24 h on (**A**) pH, (**B**) gas production (mbar), (**C**) total SCFA (mM), (**D**) acetate (mM), (**E**) propionate (mM) and (**F**) butyrate (mM). Statistical differences between the products and the NSC are indicated with * (0.01 < *p_adjusted_* < 0.05), ** (0.001 < *p_adjusted_* < 0.01) or *** (*p_adjusted_* < 0.001), while those between the products and IN are indicated with $/$$/$$$.

**Figure 3:**
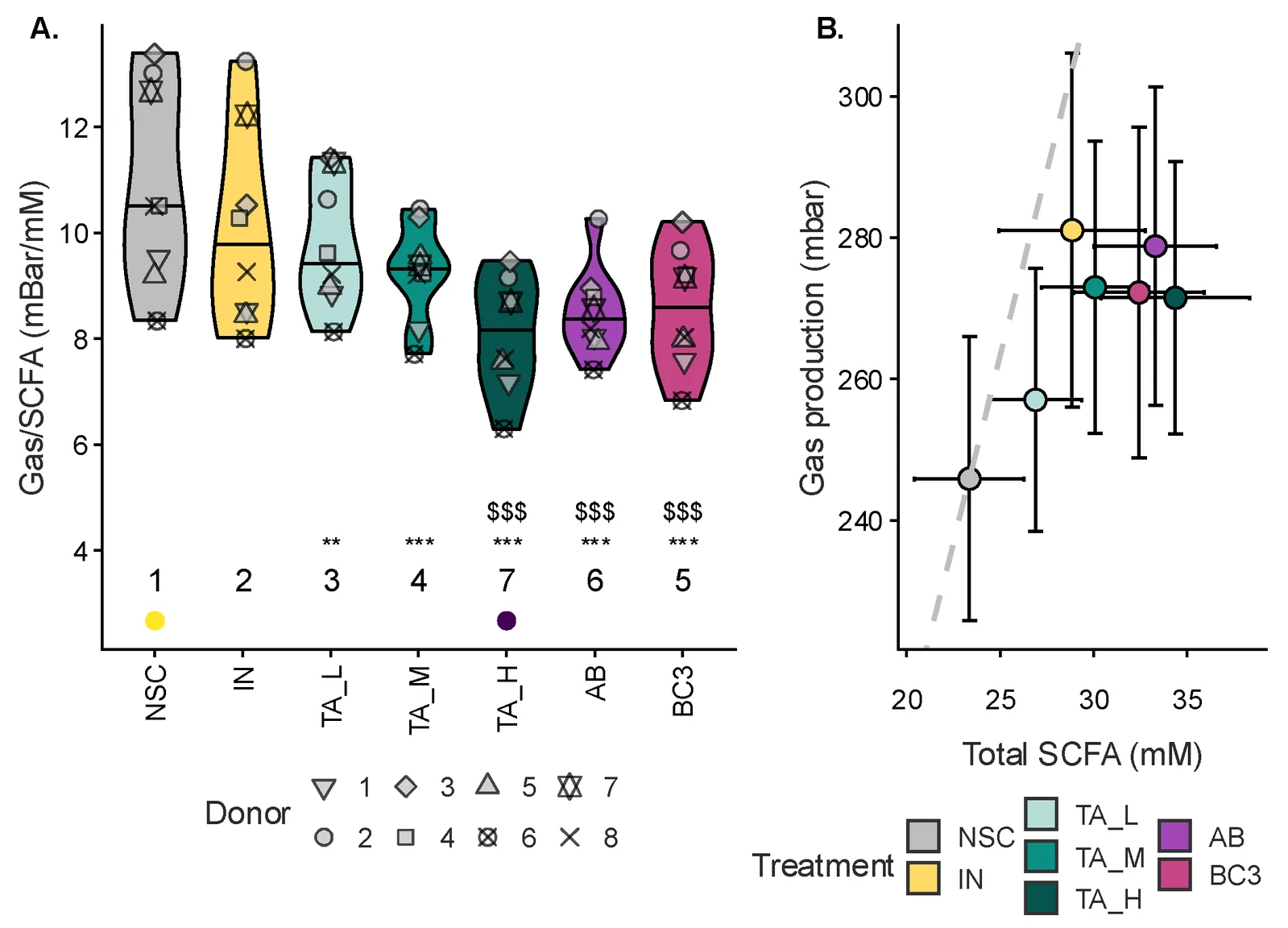
Increasing TA doses lowered the gas/SCFA ratio, with AB and BC3 exhibiting favorable profiles at 24 h. (**A**) Calculated gas/total SCFA ratio (mBar/mM) for the study arms. Statistical differences between the products and the NSC are indicated with * (0.01 < *p_adjusted_* < 0.05), ** (0.001 < *p_adjusted_* < 0.01) or *** (*p_adjusted_* < 0.001), while those between the products and IN are indicated with $/$$/$$$. (**B**) Biplot for each study arm comparing gas production (mBar) and total SCFA (mM) levels, with 95% confidence intervals shown. The dashed line indicates average the gas/SCFA ratio for NSC.

**Figure 4:**
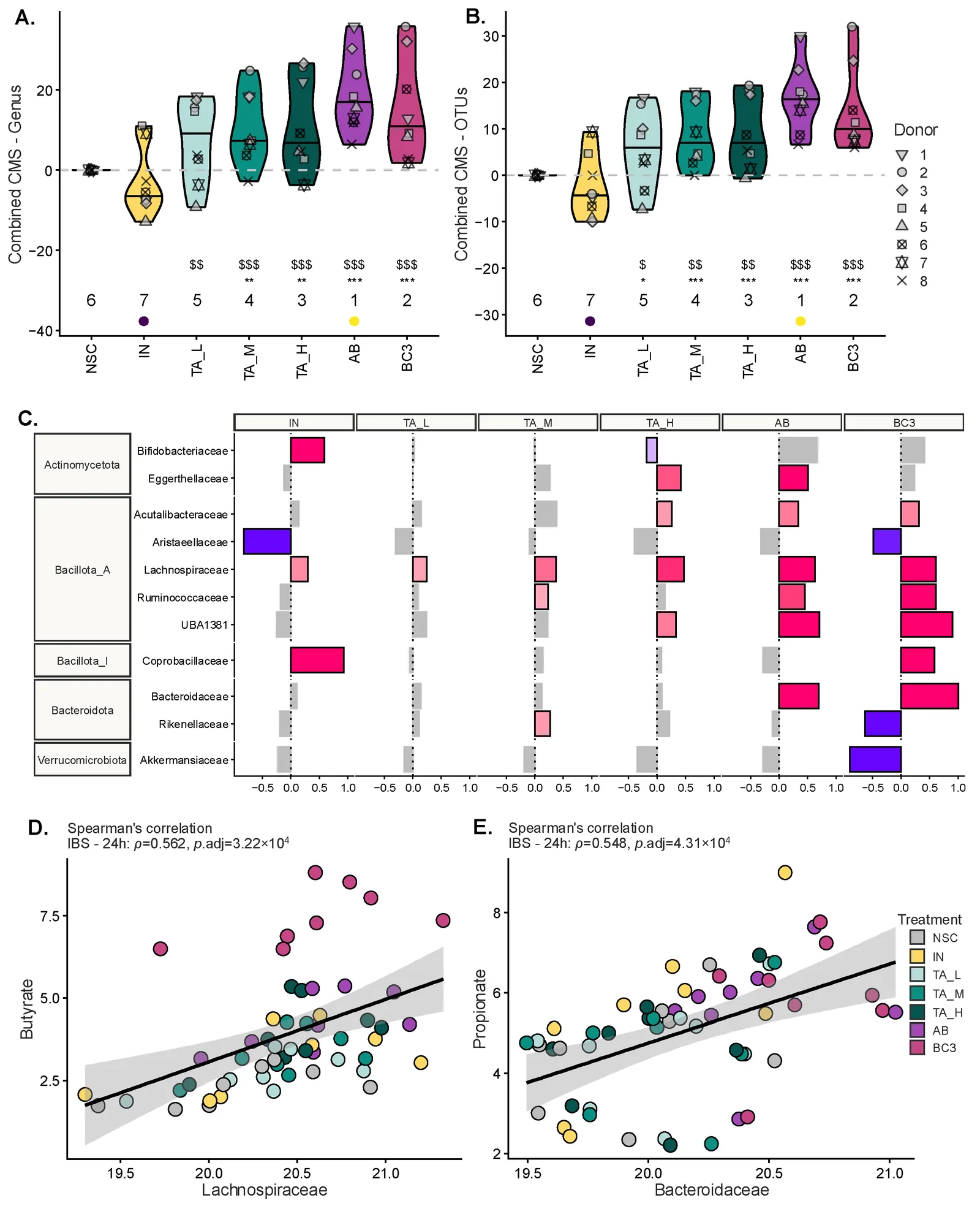
TA dose-dependently increased microbial diversity by broadly enriching butyrate-producing taxa, with AB and BC3 further modulating family-level effects. Compositional data after 24 h of colonic fermentation simulations. Statistical differences between the products and the NSC are indicated with * (0.01 < *p_adjusted_* < 0.05), ** (0.001 < *p_adjusted_* < 0.01) or *** (*p_adjusted_* < 0.001), while those between the products and IN are indicated with $/$$/$$$. (**A**) Community modulation score (CMS) at the genus level. (**B**) OTU-level CMS. (**C**) Effects of the products on relative abundance of bacterial families for the products compared to NSC, highlighted bars indicate statistical significance (*p* < 0.05). (**D**) Spearman’s rank correlation coefficient between butyrate levels and Lachnospiraceae abundance as well as (**E**) between propionate and Bacteroidaceae.

**Figure 5:**
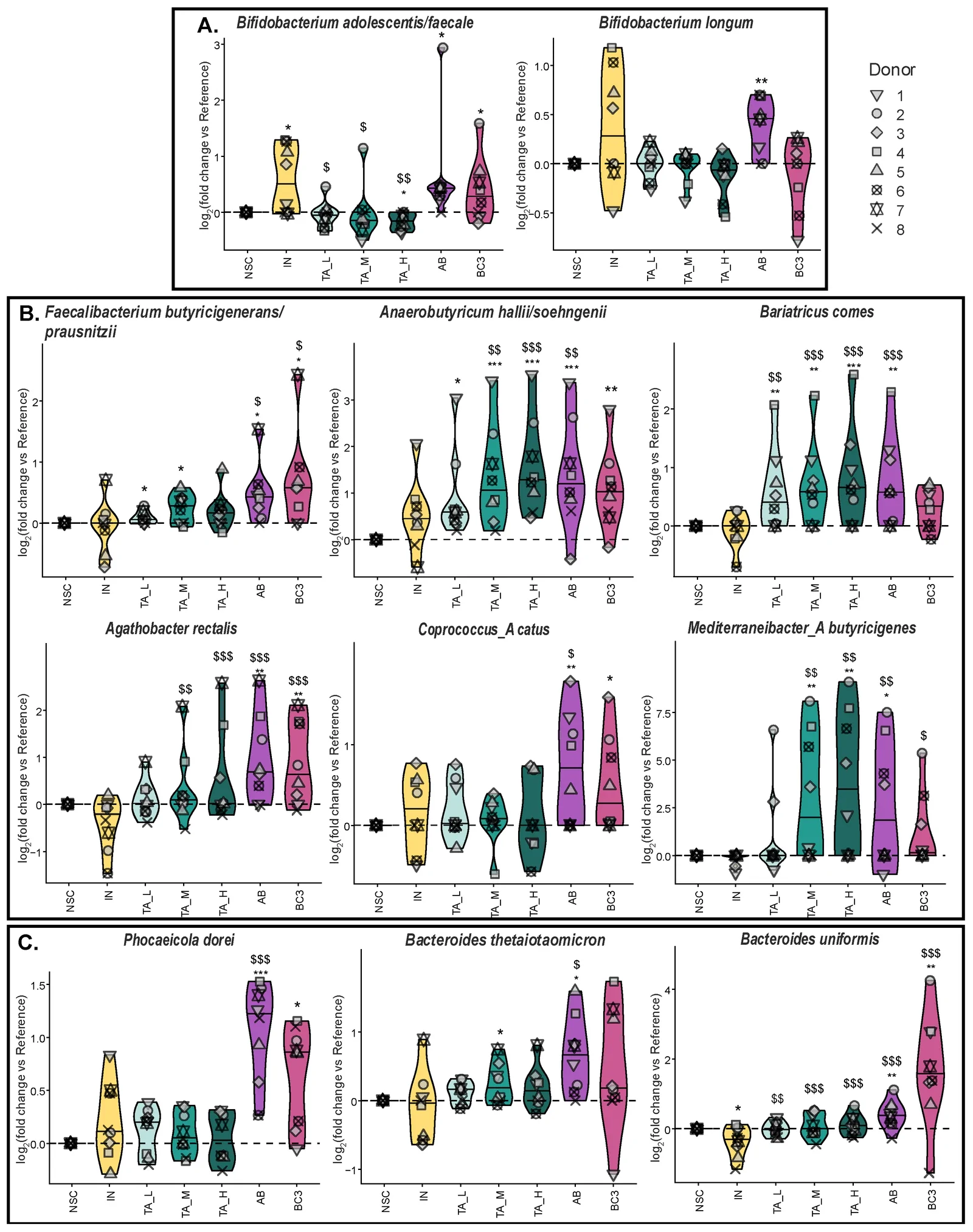
TA, AB and BC3 exhibited divergent stimulation of microbial taxa down to the OTU-level by 24 h. Impact of the products on selected taxa, represented as the log_2_-fold change in abundance compared to NSC for each donor sample. Statistical differences between the products and the NSC are indicated with * (0.01 < *p_adjusted_* < 0.05), ** (0.001 < *p_adjusted_* < 0.01) or *** (*p_adjusted_* < 0.001), while those between the products and IN are indicated with $/$$/$$$. (**A**) Bifidobacteriaceae OTUs. (**B**) Butyrate-associated OTUs within Lachnospiraceae. (**C**) Bacteroidaceae OTUs.

**Figure 6:**
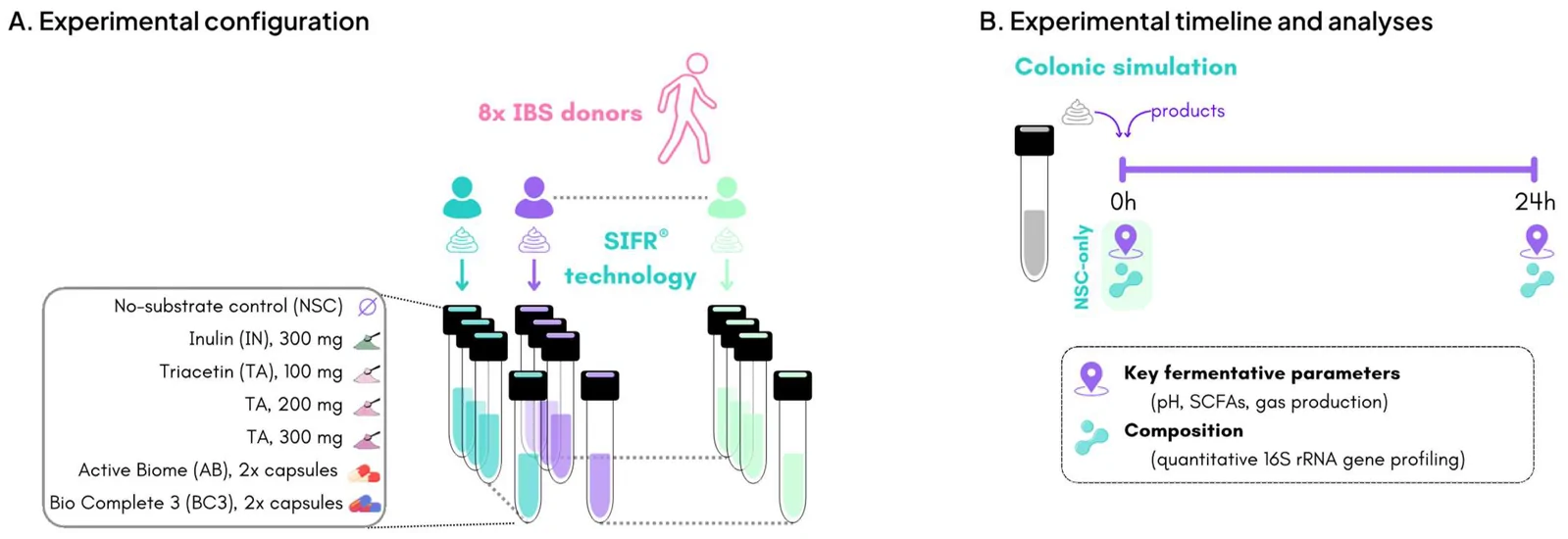
The ex vivo SIFR^®^ technology enabled mechanistic explorations of product effects on IBS-associated fecal microbiota. Study design and experimental workflow for the current work, based on *n* = 8 IBS-donor fecal samples.

## Data Availability

The raw data supporting the conclusions of this article will be made available by the authors upon reasonable request.

## Supplementary Materials

The following supporting information can be downloaded at https://www.mdpi.com/article/10.3390/ijms27198910/s1.

## Abbreviations

The following abbreviations are used in this manuscript:

AB	Active Biome™
BC3	Bio Complete 3™
CMS	Community modulation score
IBS	Irritable Bowel Syndrome
IN	Inulin
NSC	No-substrate control
OTU	Operational taxonomic unit
SCFA	Short-chain fatty acid
SIFR^®^	Systemic Intestinal Fermentation Research
TA	Triacetin
TB	Tributyrin
