# Supplementary material for: Impact of Triacetin- and Tributyrin-Based Formulations on IBS Microbiome Composition and SCFA Profiles: A Preclinical Study
Source: Int J Mol Sci. 2026 Oct 7;27(19):8910. doi: 10.3390/ijms27198910 (PMC13665126; doi:10.3390/ijms27198910)
Supplement: Supplementary file 1 [file ijms-27-08910-s001.zip › ijms-4571796-supplementary.pdf]

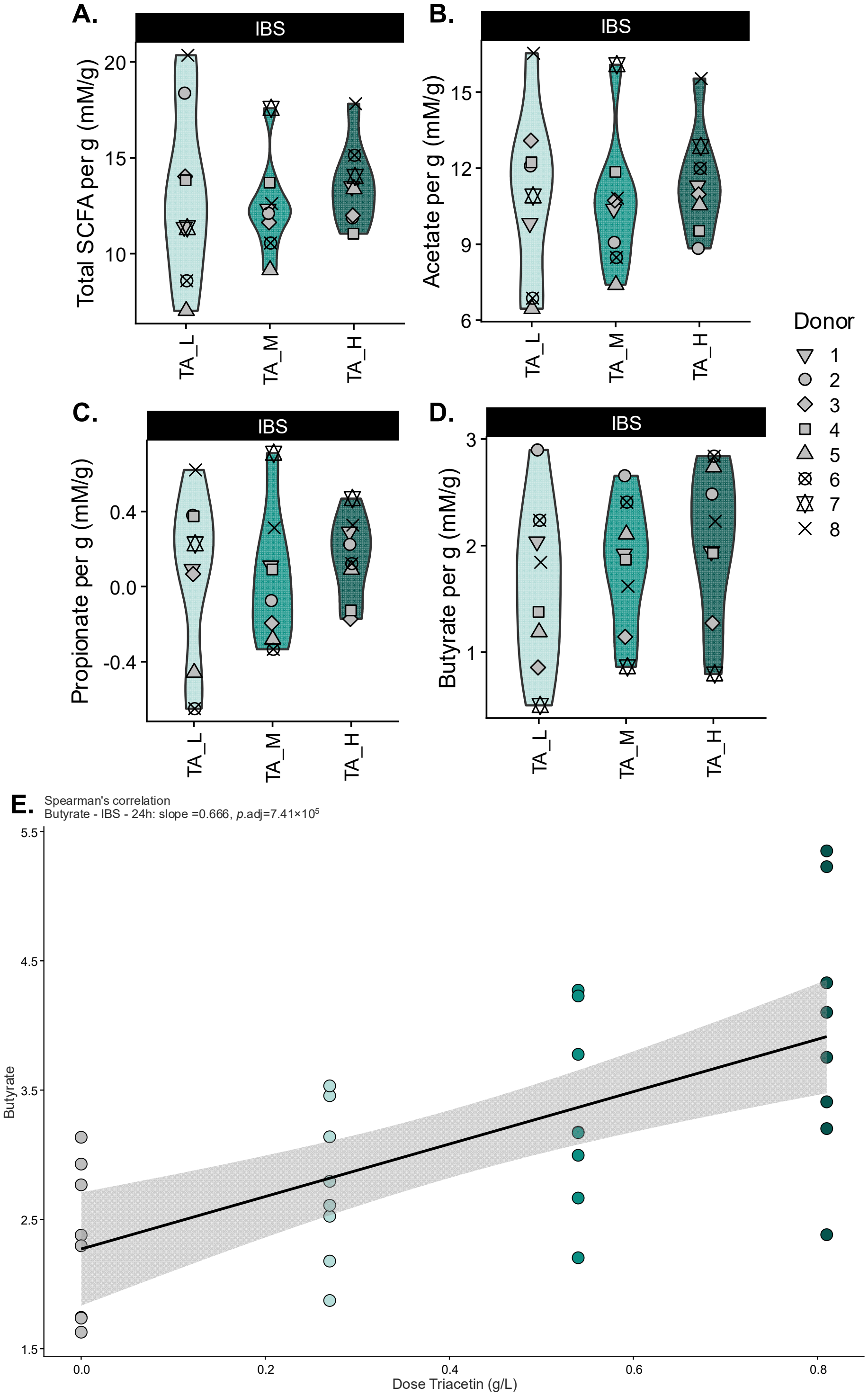

Figure S1. Triacetin (TA) doses showed dose-responsive behavior in all tested concentrations and correlated with butyrate levels. Dose-response ratios of SCFAs per TA (mM/g) at 24h, for (**A**) total SCFAs, (**B**) acetate, (**C**) propionate and (**D**) butyrate. (**E**) Spearman's correlation of butyrate levels (mM) with TA dose (g/L).

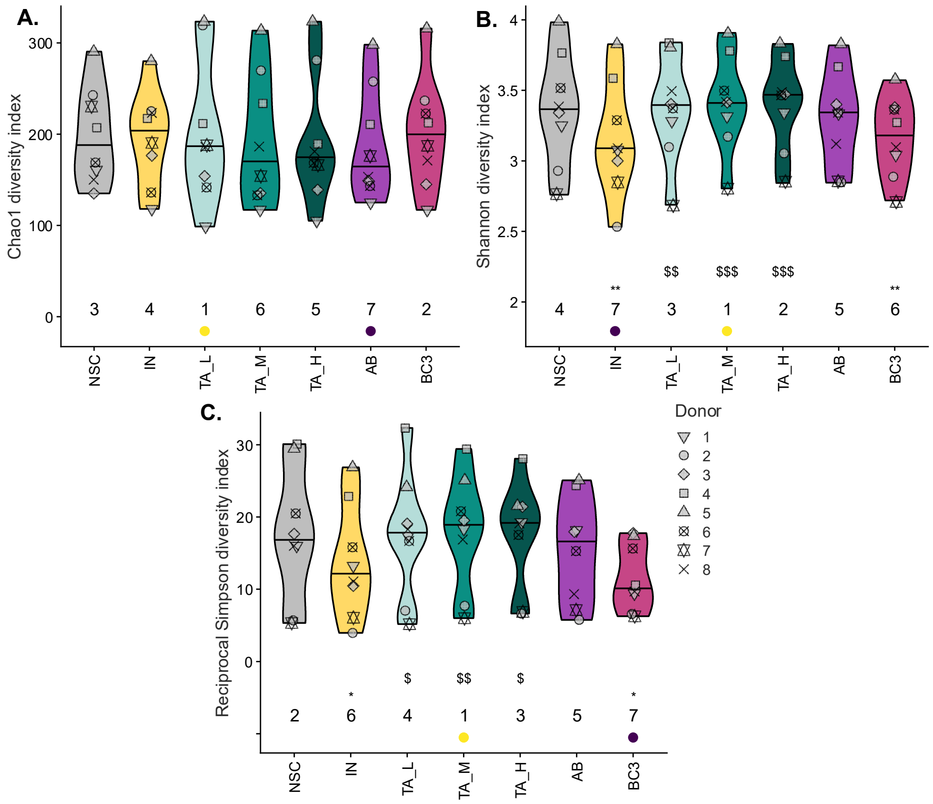

Figure S2. Impact of the products on traditional diversity indices for OTU richness and evenness. Compositional results at 24h of colonic fermentation simulations. (**A**) Chao1 index. (**B**) Shannon diversity index. (**C**) Reciprocal Simpson index.

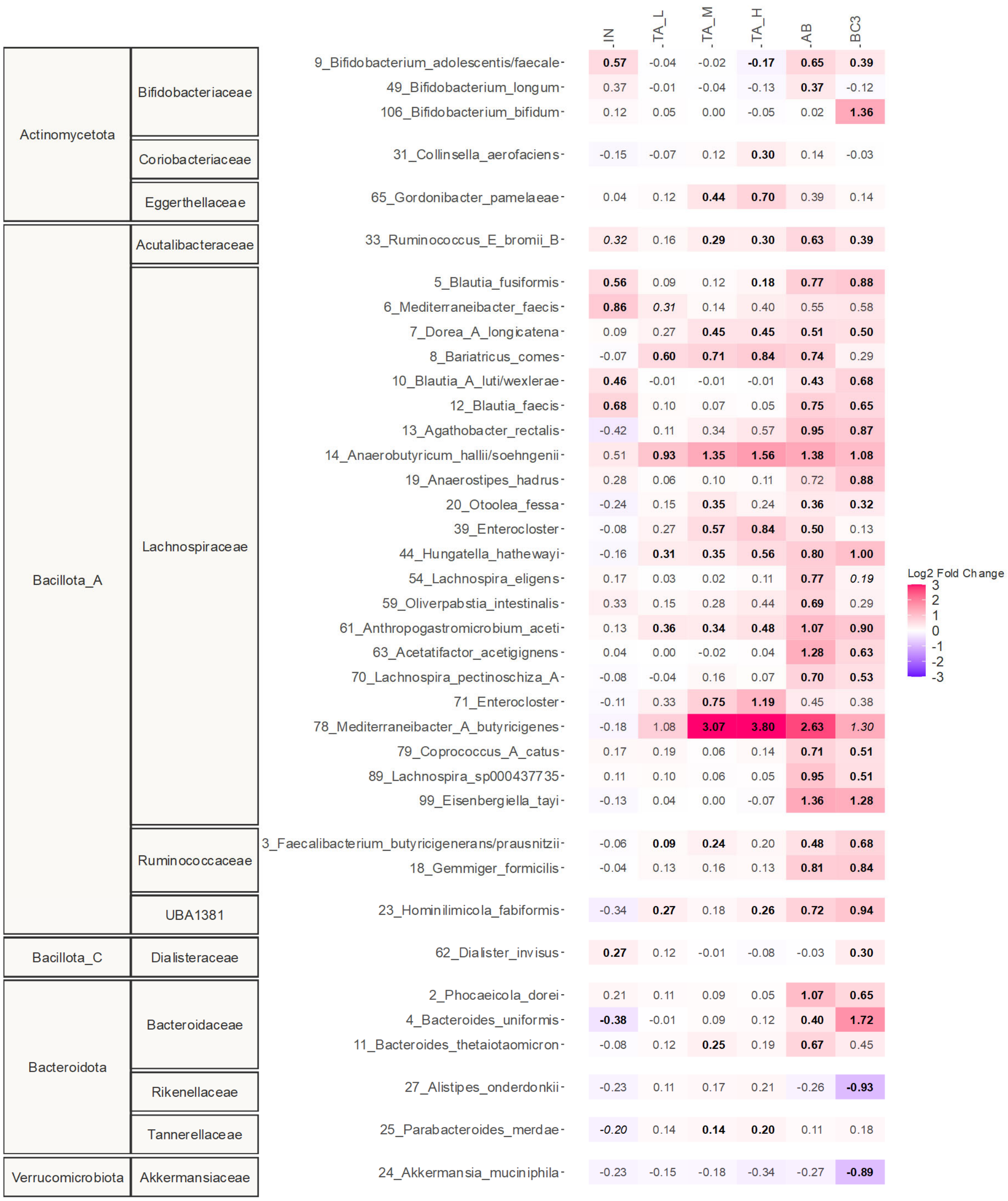

Figure S3. The products differentially impacted the IBS-associated microbiota at the OTU-level. Heatmap at the OTU-level showing the impact of the products, expressed as the log2 transformation of abundance treatment/abundance NSC ratios, averaged across all 8 donors, after 24h of colonic fermentation simulations. Significantly impacted OTUs ($p_{adjusted}$>0.05 as derived from the linear mixed model) and consistently affected OTUs (increase or decrease in at least 5/8 donors) were included. Significant effects are highlighted in bold, while those consistently yet non-significantly increased or decreased in at least 5 out of the 8 donors are shown in italics.
